# Altered Sense of Humor in Dementia

**DOI:** 10.3233/JAD-150413

**Published:** 2015-09-24

**Authors:** Camilla N. Clark, Jennifer M. Nicholas, Elizabeth Gordon, Hannah L. Golden, Miriam H. Cohen, Felix J. Woodward, Kirsty Macpherson, Catherine F. Slattery, Catherine J. Mummery, Jonathan M. Schott, Jonathan D. Rohrer, Jason D. Warren

**Affiliations:** a Dementia Research Centre, UCL Institute of Neurology, University College London, UK; b London School of Hygiene and Tropical Medicine, University of London, London, UK

**Keywords:** Alzheimer’s disease, comedy, dementia, frontotemporal dementia, humor, progressive aphasia, semantic dementia

## Abstract

Sense of humor is potentially relevant to social functioning in dementias, but has been little studied in these diseases. We designed a semi-structured informant questionnaire to assess humor behavior and preferences in patients with behavioral variant frontotemporal dementia (bvFTD; *n* = 15), semantic dementia (SD; *n* = 7), progressive nonfluent aphasia (PNFA; *n* = 10), and Alzheimer’s disease (AD; *n* = 16) versus healthy age-matched individuals (*n* = 21). Altered (including frankly inappropriate) humor responses were significantly more frequent in bvFTD and SD (all patients) than PNFA or AD (around 40% of patients). All patient groups liked satirical and absurdist comedy significantly less than did healthy controls. This pattern was reported premorbidly for satirical comedy in bvFTD, PNFA, and AD. Liking for slapstick comedy did not differ between groups. Altered sense of humor is particularly salient in bvFTD and SD, but also frequent in AD and PNFA. Humor may be a sensitive probe of social cognitive impairment in dementia, with diagnostic, biomarker and social implications.

## Introduction

Humor is a ubiquitous and highly valued social attribute and clinical experience suggests abnormalities of humor may be prominent in neurodegenerative diseases [notably, the frontotemporal lobar degenerations (FTLD)] that impair social and emotional signal decoding [1–3]. The syndromes of behavioral variant frontotemporal dementia (bvFTD) and semantic dementia (SD) are associated with impaired understanding of cartoons and sarcasm [1, 3] and this may develop premorbidly [4]. Although humor abnormalities are not generally regarded as a cardinal feature of Alzheimer’s disease (AD), emerging evidence suggests that humor might be affected alongside other aspects of social cognition in AD [5]. However, information concerning humor expression and awareness in neurodegenerative diseases remains limited.

Here we addressed this issue in patients representing canonical syndromes of FTLD [bvFTD, SD, and progressive nonfluent aphasia (PNFA)] and AD. We designed a semi-structured questionnaire to assess humor behavior and preferences, both in the current phase of established disease and retrospectively prior to clinical onset, in comparison to healthy older individuals.

## Materials AND Methods

### Participants

Forty-eight patients fulfilling current consensus criteria [6–8] for bvFTD (*n* = 15), SD (*n* = 7), PNFA (*n* = 10), or typical amnestic AD (*n* = 16) and 21 healthy older individuals (with no history of neurological or psychiatric illness, from a similar socio-cultural milieu) were recruited over a two year period from our specialist cognitive disorders clinic and from a database of healthy control research participants. Participant characteristics are summarized in Table 1. The entire patient cohort was screened for known mutations in major causative genes (*C9orf72, MAPT*, *PGRN, PS1, PS2, APP*): nine patients (eight with bvFTD, one with PNFA) with FTLD had confirmed pathogenic mutations (five *C9orf72*, four *MAPT*). Four of the five C9orf72 patients met criteria [6] for a diagnosis of bvFTD and the remaining patient was diagnosed with PNFA according to established criteria [7].

Syndromic diagnoses were further corroborated in all cases by neuropsychological assessment, brain imaging (CT in three patients, MRI in the remainder), and/or cerebrospinal fluid examination. Participants had a comprehensive general neuropsychological assessment including standard measures of executive (Wechsler Abbreviated Scale of Intelligence, WASI) [9], social cognitive (The Assessment of Social Inference Test, TASIT) [10], semantic (British Picture Vocabulary Scale, BPVS) [11], and visual perceptual functions (see Table 1). All patients had neuroimaging findings compatible with their clinical syndromic diagnosis (corresponding to a diagnosis of ‘probable bvFTD’) in the bvFTD group [6]. Cerebrospinal fluid tau and Aβ_1-42_ assays in 20 patients (six bvFTD, six PNFA, eight AD) supported the clinical diagnosis in all cases, based on local laboratory reference ranges (normal ranges; total tau <320, Aβ_1-42_ 220–2000, tau/Aβ_1-42_ ratio >0.8 predictive of AD). Of the genetic cases, four also had cerebrospinal fluid data available, confirming a non-AD-likeprofile.

The study was approved by the local institutional ethics committee and all participants gave written informed consent following Declaration of Helsinki guidelines.

### Humor questionnaire

In order to assess patients’ sense of humor in daily life, we designed a semi-structured questionnaire comprising seven items (Table 2). Questionnaires were completed for each patient by a normal informant who had known them well for at least 15 years. The questionnaire recorded perceived changes in the patient’s sense of humor over the course of the illness and an item adapted from the Cambridge Behavioural Inventory (CBI) was used to quantify any tendency to express humor in scenarios that others would not generally find funny (rated 0–4; 0 = never, 1 = a few times per month, 2 = a few times per week, 3 = daily, 4 = constantly). In addition, the questionnaire recorded patients’ total daily life comedy exposure in broadcast and print media (estimated hours per week) and their liking for comedy (on a 10-point Likert scale), both currently and 15 years previously. This interval was chosen arbitrarily, but was designed to capture any alterations in humor preferences before the onset of typical clinical symptoms, while minimizing potential confounding effects from normal cognitive aging, informant knowledge, and recall bias. Patients with disease duration longer than 15 years were accordingly not included in the study. The questionnaire assessed three broad comedy genres or categories; farcical or slapstick (e.g. *Mr Bean*), satirical (e.g. *Yes, Minister*) and absurdist (e.g. *Monty Python*). The questions on comedy exposure and preferences were also administered to healthy older participants (accordingly, questionnaire data for the healthy control group were based on self-report).

### Statistical analyses

Demographic characteristics, neuropsychological and behavioral rating data were compared between groups. Data on participant gender, country of origin and altered sense of humor (present/absent) were analyzed using two-tailed Fisher’s exact tests. Kruskal Wallis tests were used to compare other demographic characteristics, comedy exposure and liking for particular comedy genres between groups. Relations between humor preference ratings and gender were assessed in the healthy control cohort using the Wilcoxon rank-sum test. Spearman’s tests were used to assess correlations of humor measures with general disease measures (symptom duration, Mini-Mental State Examination (MMSE) score) and nonverbal executive performance (WASI Matrices score) in the combined patient cohort, semantic performance (BPVS score) in the SD group and social cognitive performance (TASIT scores) in the bvFTD group. For all tests, *p* <  0.05 was accepted as the threshold of statistical significance.

## Results

Participant groups did not differ significantly in mean age (*p* = 0.54) or education (*p* = 0.25; see Table 1). Males were significantly over-represented in the bvFTD group compared with the healthy control group (*p* = 0.04); gender was not significantly correlated with any humor measure (all *p* >  0.05) in the healthy control reference group and accordingly was not analyzed further. Patient groups did not differ in estimated symptom duration (*p* = 0.77); the AD group had a significantly lower MMSE score than the bvFTD group (*p* = 0.03).

Humor questionnaire data are summarized in Table 3 and representative informant comments are in Table 4. Three patients with bvFTD— one with a pathogenic C9orf72 mutation, one with a MAPT mutation, and one with no identified mutation on screening— were not entered into the study because estimated symptom duration was >15 years in these cases. In each case, the patient’s caregiver described alterations in their sense of humor similar to other patients with bvFTD. Questionnaire informants were mainly the patients’ primary caregivers (in most cases, a cohabiting spouse) or a sibling or child who had been in long-term regular contact (at least monthly) with the patient (Table 3). Most participants had grown up in the United Kingdom; a few had spent part of their childhoods abroad in countries affiliated with Britain (Table 3). One patient with bvFTD was excluded owing to the fact he was a French national (and had therefore experienced a comedy milieu not shared by the rest of the cohort). Participant groups did not differ significantly according to country of origin (*p* = 0.62;see Table 3).

Altered sense of humor was reported significantly more frequently in bvFTD (*p* <  0.01) and SD (*p* <  0.05) (all patients) than PNFA or AD (around 40% of patients). Patients with bvFTD were significantly more likely to express humor in situations not generally considered humorous than patients with SD or PNFA (*p* <  0.01; borderline significant versus AD, *p* = 0.051). Other patient groups did not differ with respect to expressed humor.

The CBI measure of increased tendency to show humor was significantly correlated with executive impairment (WASI Matrices) for the combined patient cohort (rho = –0.36, *p* = 0.018), but additionally correlated with symptom duration only in AD (rho = 0.62, *p* = 0.014). No other significant within-group correlations were identified between humor measures and general disease severity or executive performance measures (all *p* >  0.05). In the SD group, no humor measure showed a significant correlation with semantic performance as assessed using BPVS score (all *p* >  0.05). In the bvFTD group no humor measure showed a significant correlation with social cognitive performance as assessed from TASIT scores (total score, emotion subscore, sarcasm subscore all *p* >  0.05).

Informant comments (Table 4) revealed a number of instances in which patients were reported to show frankly inappropriate humor responses such as laughter over others’ misadventure (e.g. watching news stories about natural disasters, witnessing a spouse injure herself) or impersonal stimuli (e.g. a car badly parked, a barking dog). In a *post hoc* analysis, such inappropriate humor responses were significantly over-represented in bvFTD (*p* <  0.01) and SD (*p* <  0.05), occurring in around half these patients, but not at all in PNFA or AD. Informant reports indicated a shift in patients’ comedy preferences toward the fatuous and farcical as the clinical syndrome became established (Table 4). Estimated overall comedy exposure (hours/week) did not differ significantly between participant groups either currently (*p* = 0.07) or premorbidly (*p* = 0.24; Table 3). However, current liking for satirical and absurdist comedy was significantly less in all patient groups compared with healthy controls (*p* <  0.05) and liking for satirical comedy (though not other comedy genres) was significantly less in bvFTD and AD compared with PNFA (*p* <  0.05). Premorbidly, liking for satirical comedy was significantly less in bvFTD, PNFA, and AD (though not SD) compared with healthy controls (*p* <  0.05). This change was estimated to have been evident between two to 13 years (on average, at least nine years) prior to onset of more typical symptoms. Patient groups did not differ premorbidly in their liking for satirical comedy and no patient group showed premorbid alterations in liking for other comedy genres.

**Fig.1:**
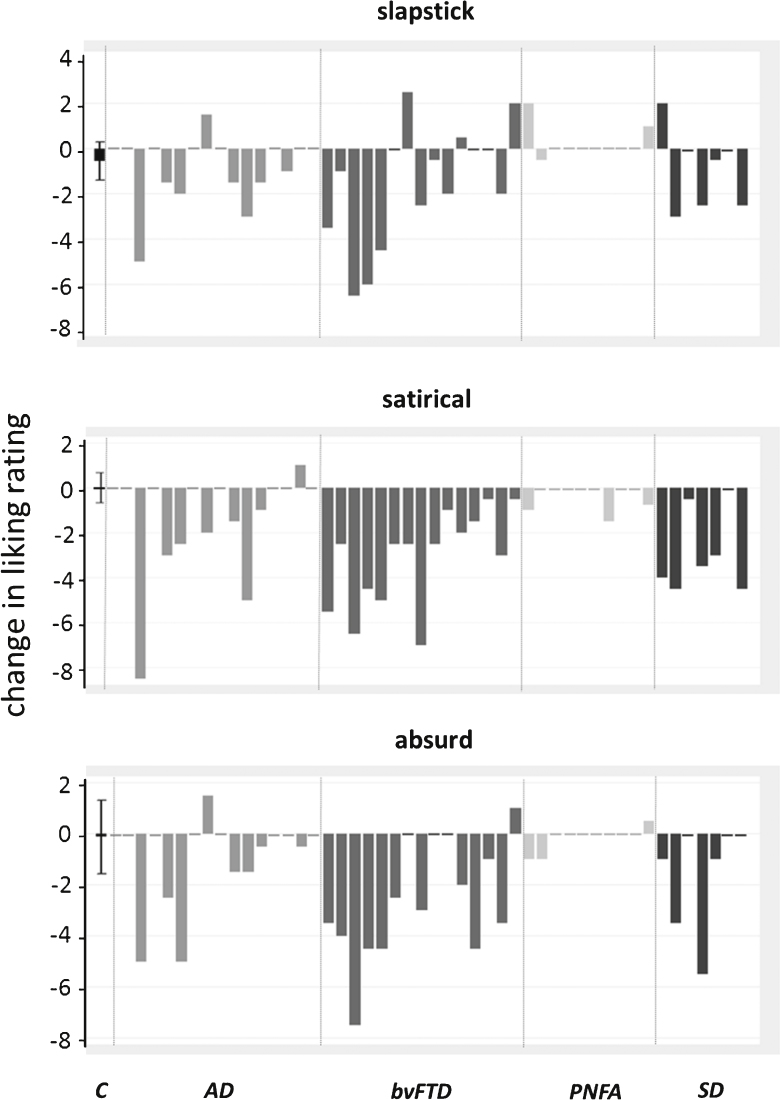
Questionnaire data on changes in liking of comedy over a 15 year interval are shown for individual patients in each disease group (Alzheimer’ disease, AD; behavioral variant frontotemporal dementia, bvFTD; progressive nonfluent aphasia, PNFA; semantic dementia, SD) alongside the mean change in liking for the healthy control group (C), with error bars indicating standard deviation from the mean in controls. Data for each comedy genre are plotted in separate panels. In each plot, the zero line indicates no change over the interval; values below the line indicate reduced liking and values above the line increased liking for that comedy genre, on a 10-point Likert scale (see text and Table 2 for details).

Questionnaire data on liking for particular comedy genres in individual patients are presented in Fig. 1. These data show that the majority of patients with bvFTD and SD showed reduced liking for comedy, while most patients with PNFA showed no change in liking for comedy across genres following the onset of their illness. However, a few patients in each group showed increased liking for comedy; this occurred most frequently for slapstick comedy and in patients with bvFTD and PNFA (20% of patients in each of these groups).

*Post-hoc* analyses of genetic bvFTD subgroups revealed no differences with respect to any humor characteristic compared with the sporadic bvFTD subgroup. One patient with predominant right temporal lobe atrophy was included in the cohort; this patient had a profile of humor alterations that was qualitatively similar to other bvFTD cases.

## Discussion

Here we have shown that canonical dementia syndromes commonly produce an altered sense of humor and this alteration differs qualitatively and quantitatively between dementia syndromes. In this series, altered humor was universal in bvFTD and SD, but occurred in a substantial minority of patients with PNFA and AD. Increased fatuity and relative predilection for childlike or slapstick humor and less pleasure in other comedy genres were features of all dementia syndromes, while frankly inappropriate humor in response to unpleasant or impersonal stimuli was a hallmark of bvFTD and SD. Moreover, selectively altered humor responsiveness was reported to have occurred well before the onset of more typical symptoms in association with both FTLD and AD: this was manifest as less pleasure in satirical comedy premorbidly. Development of abnormal humor expression correlated with executive impairment across syndromes and with clinical disease duration in AD, but not FTLD syndromes; supporting the clinical impression that sense of humor is often impoverished early in FTLD, but relatively preserved initially in AD.

The most striking alterations of humor responsiveness here occurred in FTLD syndromes characterized by impaired interpersonal functioning and for comedy genres (satirical, absurdist) most reliant on social cognition processes. This corroborates recent cognitive profiling of these syndromes using a novel paradigm assessing patients’ perception of humor in nonverbal cartoons [12]: both bvFTD and SD were associated with impaired detection of humorous intent in cartoon scenarios requiring psychological insight. Whereas the appreciation of slapstick humor typically entails detection of surface and physical incongruities, appreciation of satirical and absurd comedic scenarios requires a model of our place in the world with an understanding of social norms and often, others’ beliefs and intentions (‘theory of mind’ [1]). Transgression of those norms or mental states can then be perceived as surprising and solving the puzzle this poses, as ultimately pleasurable (when we ‘get’ the joke [13]). In patients with bvFTD and SD, this puzzle-solving behavioral algorithm appears to be not simply defective, but promiscuous: such patients are apt to assign humorous value in highly inappropriate contexts.

This interpretation would align an altered sense of humor with other forms of aberrant reward processing in FTLD [14], further demonstrating that abnormal valuation can extend from primary biological reinforcers such as food and sex to complex, abstract sensory stimuli [15] and potentially reflecting shared mechanisms of abnormal reward decoding in striatal and mesolimbic brain networks. The lack of correlation here between humor measures and a standard measure of social cognitive function (TASIT) in the bvFTD group might therefore appear initially somewhat counterintuitive; while arguments to a negative finding must be cautious particularly in the face of small case numbers, this might reflect the modularity of social cognitive subcomponents and suggests that substrates for humor decoding may be at least partly separable from other social cognition processes [12]. It is also noteworthy that only a minority of patients with bvFTD were reported as showing enhanced liking for slapstick comedy (Fig. 1) despite a clear tendency to increased fatuity and inability to suppress humor responses. This might indicate that humor behaviors in these patients become ‘mirthless’ (dissociated from subjective pleasure) or alternatively, that the behavioral correlates of such pleasure are harder for normal informants to decode. Further in this regard, our finding that sense of humor is commonly altered in AD is somewhat surprising and should motivate further study. The cognitive basis of this alteration may differ fundamentally in AD and FTLD: in particular, humor alterations in AD might reflect over-identification with the plight of others, as a manifestation of eroded emotional boundaries [5].

This study has several limitations that should guide future work. Clinical group sizes here were relatively small and patients were assessed using third-person reports while control data were based on self-report, both potentially subject to recall bias. There is a need to validate the questionnaire we propose in future work and in participants from other ethnic and cultural backgrounds. Humor is heavily influenced by social and cultural context and assessment of humor appreciation will likely require tailoring to these factors. Humor behavior and its potential as a disease biomarker should be studied prospectively, ideally from the presymptomatic phase of genetically-mediated dementias and with direct autonomic, structural and functional neuroanatomical correlation to capture subjective alterations in the experience of humor and mirth. Ideally, humor should also be assessed in the setting of neurological disease that spares cognitive function, in order to disambiguate cognitive from nonspecific chronic disease effects. The nature of humor alterations in neurodegenerative diseases requires further clarification: humor abnormalities have probably been under-recognized in AD and their relation to poorly understood phenomena such as abnormal laughter in PNFA [16] remain to be clarified. In particular, there is a need to investigate in greater detail the relations between humor alterations and other components of social cognition in these diseases. More broadly, the present findings have implications for the social functioning and quality of life of patients and those who care for them and this should be explored explicitly. We hope that our findings will stimulate interest in humor as an engaging, ecologically relevant and informative index of social functioning in neurodegenerative disease.

## Figures and Tables

**Table 1:** General demographic, clinical and neuropsychological characteristics of participant groups

Characteristic	bvFTD	SD	PNFA	AD	Controls
**GENERAL**
No. (M:F)	**13:2**	4:3	5:5	8:8	11:10
Age (y)	65 (7.3)	66.9 (6.2)	69.4 (7.4)	66.1 (8.0)	65.9 (5.0)
Education (y)	15 (2.6)	14 (2.4)	16 (2.5)	14 (2.9)	16 (1.9)
Symptom duration (y)	6.3 (3.4)	5.7 (3.3)	5.1 (2.6)	6.1 (2.7)	N/A
MMSE (/30)	25 (4)	22 (8)	21 (10)	20 (5)^a^	N/A
**BACKGROUND NEUROPSYCHOLOGY**
**General intellect**
VIQ	**85 (21)**	**76 (19)^b^**	**82 (19)**	**93 (22)**	123 (6)
PIQ	**96 (14)**	**109 (23)**	**98 (21)**	**85 (18)^c^**	126 (10)
WASI Vocabulary (/80)	**42 (21)**	**35 (22)**	**42 (17)**	**53 (17)**	71 (4)
WASI Block Design (/71)	**25 (15)**	**35 (20)**	**21 (17)**	**14 (14)^a,c^**	51 (10)
WASI Similarities (/48)	**25 (13)**	**22 (13)**	**28 (7)**	**25 (11)**	42 (3)
WASI Matrices (/42)	**17 (7)^c^**	23 (8)	**21 (6)**	**12 (7)^a,c,d^**	27 (3)
**Executive**
Stroop (ink color) (s)	**100 (41)**	**89 (50)**	**140 (33)**	**118 (47)**	54 (11)
Trails (B-A difference) (s)	**131 (91)**	78 (76)^d^	**150 (58)**	**130 (84)**	36 (24)
**Social cognition**
TASIT emotion (/14)	**8.3 (2.6)**	N/A	N/A	N/A	12 (1.3)
TASIT social inference (/36)	**22 (6.0)**	N/A	N/A	N/A	31 (2.2)
**Language**
GNT (/30)	13 (8)	**3 (4)^a,b,d^**	**18 (7)**	**16 (9)**	28 (2)
BPVS (/150)	136 (14)	**97 (49)**	**142 (9)**	**119 (51)**	148 (2)
Reading (NART) (/50)	31 (14)	**24 (21)**	**34 (10)**	**29 (13)**	44 (3)
**Episodic memory**
RMT Words (Z score)^ *^	–1.3 (1.3)	–1.5 (1.5)	–1.1 (1.4)	–1.6 (0.9)	0.6 (0.2)
RMT Faces (Z score)^ *^	–1.9 (1.1)	–0.6 (1.2)	0.4 (0.3)	–1.9 (0.9)	0.2 (0.7)
**Other skills**
WMS-R digit span forward (/12)	8.4 (2.3)	9.4 (2.4)	7.6 (1.6)	**6.3 (2.5)^a,c^**	8.9 (2.0)
WMS-R digit span reverse (/12)	6.5 (2.2)	8.4 (2.9^) ^	**4.1 (2.7)^c^**	**6.3 (5.3)**	7.3 (1.9)
GDA (/24)	**10 (6.5)**	11 (9.7)	**5 (4.8)**	**11 (13.3)**	15 (4.4)
VOSP Object Decision (/20)	17 (1.9)	18 (2.4)	17 (2.8)	16 (3.8)	19 (1.7)

Mean (standard deviation) data are presented unless otherwise indicated. Maximum neuropsychological test scores are in parentheses. Bold denotes significantly different from healthy controls, *p* <  0.05; ^ *^floor performance –2.67 from age norms (long RMT) except AD floor performance –1.88 (short RMT); ^a^significantly different from bvFTD; ^b^significantly different from AD; ^c^significantly different from SD; ^d^significantly different from PNFA; AD, Alzheimer’s disease; BPVS, British Picture Vocabulary Scale [11]; bvFTD, behavioral variant frontotemporal dementia; D-KEFS, Delis Kaplan Executive System [17]; GDA, Graded Difficulty Arithmetic [18]; GNT, Graded Naming Test [19]; MMSE, Mini-Mental State Examination score [20]; N/A, not assessed/available; NART, National Adult Reading Test [21]; PIQ, performance IQ; PNFA, progressive nonfluent aphasia; RMT, Recognition Memory Test [22]; SD, semantic dementia; Trails-making task (B-A difference) scored on maximum of 2.5 minutes on task A, 5 minutes on task B [23]; VIQ, verbal IQ; VOSP, Visual Object and Spatial Perception Battery [24]; WASI, Wechsler Abbreviated Scale of Intelligence [9]; WMS-R, Wechsler Memory Scale Revised [25].

**Table 2:** Questionnaire to assess patients’ daily life humor preferences

Daily Life Humour Questionnaire
1. Care-giver’s relationship to patient:
2. How long have you known the patient? (years):
3. What country did s/he mainly grow up in? (to age 16)
4. Has s/he exhibited a change in sense of humour in the course of the illness?’
If so, in what way?
5. Does s/he find humour or laugh at things others do not find funny? Please rate:
0, never; 1, a few times per month; 2, a few times per week; 3, daily; 4, constantly
6. Please estimate the total hours in a typical week that s/he spends watching comedy programmes (TV or films) or looking at humorous cartoons:
Currently:	15 years ago:
7. Please rate his/her liking for comedy of the following kinds, according to the scale shown below
1	10
Dislikes very much	Likes very much
7.1 Slapstick or farcical comedy, e.g. Mr Bean, Benny Hill, Tom and Jerry
Currently:	15 years ago:
7.2 Satirical comedy, e.g. Yes, Minister, Punch, The New Yorker
Currently:	15 years ago:
7.3 Absurdist comedy, e.g. Monty Python, The Goon Show
Currently:	15 years ago:

The questionnaire was completed by a normal informant for each patient, in most cases their primary caregiver; healthy control participants completed a modified version of the questionnaire (comprising Questions 3,6 and 7). Informants were encouraged to seek clarification on examples of comedy genres to improve reliability and avoid bias. Patients were all known to their primary informants for >15 years.

**Table 3:** Humor questionnaire data for the participant groups

Characteristic	bvFTD	SD	PNFA	AD	Healthy controls
***Background***
Informant’s relationship to patient (spouse:other)	13:2^ ¥^	5:2^*α*^	7:3^β^	15:1^*γ*^	N/A
Average duration of relationship (y)	44.7 (11.5)	40.1 (9.1)	44.2 (9)	43.4 (10.6)	N/A
Participant country of origin (UK/Eire: other)	15:0	6:1^ †^	10:0	15:1^ †^	19:2^ ‡^
***Humor: Over course of illness***
Altered sense of humor? (Y:N)	15:0^a^	7:0^b^	4:6	7:9	NA
Inappropriate humor (Y:N)^¶^	8:7^c^	4:3^d^	0:10	0:16	NA
Tendency to laugh: frequency^¶¶^	1.8 (1.2)^e^	0.4 (0.2)	0.1 (0.3)	1 (1.4)	NA
***Humor: Currently***
Total comedy exposure†(h/wk)	5.8 (13.3)	0.4 (0.6)	2.1 (1.7)	1.6 (1.7)	1.5 (1.2)
Liking††: slapstick	4.1 (2.8)	3.6 (2.0)	4.5 (2.5)	3.7 (1.6)	4.9 (2.1)
Liking: satirical	**3.1 (1.7)**	**4.1 (3.7)**	**5.9 (1.9)^f^**	**3.8 (1.8)**	7.7 (1.5)
Liking: absurd	**3.3 (2.5)**	**3.5 (3.2)**	**4.6 (2.7)**	**4.1 (2.0)**	6.3 (2.1)
***Humor: 15 years ago***
Total comedy exposure (h/wk)	5.4 (7.1)	2.8 (2.8)	1.8 (1.4)	2.7 (2.0)	3.3 (2.6)
Liking: slapstick	5.7 (2.0)	4.5 (2.3)	4.3 (2.0)	4.5 (1.9)	5.5 (2.1)
Liking: satirical	**6.2 (1.9)**	7.0 (2.1)	**6.2 (1.8)**	**5.2 (2.2)**	7.7 (1.4)
Liking: absurd	5.9 (2.2)	5.0 (3.4)	4.7 (2.7)	5.1 (2.4)	6.4 (2.2)

Mean (standard deviation) values are shown unless otherwise indicated; bold denotes significantly different from healthy control group (thresholded at *p* = 0.05). ^ ¥^two siblings; ^*α*^two children, ^β^two children, one friend ^*γ*^one child; ^ †^one participant grew up in South Africa; ^ ‡^one participant grew up in Canada, one participant was subsequently found to have been brought up in Denmark:^¶^ based on *post hoc* analysis of informant reports (see text); ^¶¶^ from Cambridge Behavioural Inventory (data available for 15 patients with bvFTD, six patients with SD, nine patients with PNFA, 15 patients with AD), scaled as: 0 (never), 1 (a few times a month), 2 (a few times a week), 3 (most days) or 4 (constantly); ^ †^broadcast and print media; ^ ††^10 point Likert scale (1, dislikes very much to 10, likes very much); ^a^significantly different from PNFA (*p* = 0.001) and AD (*p* = 0.001); ^b^significantly different from PNFA (*p* = 0.035) and AD (*p* = 0.019); ^c^significantly different from PNFA (*p* = 0.008) and AD (*p* = 0.001); ^d^significantly different from PNFA (*p* = 0.015) and AD (*p* = 0.004); ^e^significantly different from SD (*p* = 0.004) and PNFA (*p* = 0.0004), borderline significantly different from AD (*p* = 0.051); ^f^significantly different from bvFTD (*p* = 0.002) and AD (*p* = 0.02). Alzheimer’s disease; bvFTD, behavioral variant frontotemporal dementia; PNFA, progressive nonfluent aphasia; SD, semantic dementia.

**Table 4:** Representative informant comments recording instances of altered humor exhibited by patients (case identifier numbers are used here for convenience only); references to inappropriate humor are in bold

Case	Group	Informant comment
1	bvFTD: C9orf72	**Has developed a dark and misplaced sense of humour; relishes other people’s mishaps or upset**
2		Rarely laughs heartily at a joke like before. Tells a filthy joke, wonders why others don’t laugh
3		Previous dry and entertaining sense of humour has completely disappeared; rarely laughs now
4		Still sees humour in some things- particularly those of a more visual nature (eg slapstick); **will laugh at things inappropriately eg. after messy eating;** inclined to mimic others who smile or laugh
5	bvFTD: MAPT	Very rarely laughs these days, **laughs when see a disaster on the news**
6		Rarely laughs at jokes now except own, **often inappropriately.** Jokes taken literally, misses the point
7		Used to be very witty but that has all gone; humour has to be more obvious, laughs if others laugh
8		Almost zero sense of humour
9	bvFTD: sporadic	Idea of humour now very rude and graphic, everything is now ‘funny’
10		Was very sharp and clever with words, now finds slapstick/childlike humour very funny; **frequently laughs at a disaster on the news or a child falling off their bike**
11		Early on laughed very loudly at things that were only mildly funny, flippant or ‘over the top’; now laughs all the time at things that are not particularly funny and will say “I’m laughing and I’m not sure why I’m laughing”. **When I badly scalded myself the other year, thought it was hilarious**
12		Has little sense of humour at all, does not really find anything funny but will give a silly laugh or sneer **when totally inappropriate.** Does not find any humour in our new puppy
13		Tends not to laugh as much at things previously thought funny (e.g. *Dad*’*s Army)*, **sometimes laughs inappropriately at news items**
14		Has always been a joker, but this has increased- **not always appropriately**
15		Cannot understand nuances, irony
16	SD	Sense of humour now simpler, or more basic, no longer comprehends complex jokes, more likely to laugh at slapstick comedy or **things that seem out of place (e.g. car parked on pavement), coincidences**
17		Doesn’t seem to know when someone is joking and tends to take everything at face value
18		Much more likely to make ‘silly’ comments (eg. “it won’t suit you” if I say “I’ll put the kettle on”)
19		Now rarely laughs unless more obvious, slapstick humour, but no longer e.g. *Monty Python*; **often laughs at things that are not funny, e.g. personal misfortune** and TV programmes used to find puerile
20		Now virtually devoid of humour; cannot appreciate word based jokes or visually based jokes, will laugh if others are laughing or things that **aren’t funny, e.g a barking dog**
21		Doesn’t get subtleties, e.g. used to read *Private Eye*, but now needs jokes explained
22		**I have asthma - laughs sometimes when I am fighting to get my breath**
23	PNFA	More keen on slapstick and farce
24		Laughs more at black humour but less into comedy
25		Sometimes laughs at things others don’t
26		More childish and immature; laughs in a loud and embarrassing way^ *^
27	AD	Makes several “non” jokes per day, mostly verbal plays and puns, compulsive
28		Now finds childish humour funny
29		Does not tell as many jokes as before, more smutty humour
30		Doesn’t laugh very often, humour needs to be very simplistic
31		Doesn’t understand jokes even when explained, may become angry when others laugh at something
32		A bit more vulgar, will tell jokes that really aren’t funny, laughs at own remarks a lot.
33		Slower to detect humour as looks for literal meaning, less humour than before

^ *^This patient had a C9orf72 mutation. AD, Alzheimer’s disease; bvFTD, behavioral variant of frontotemporal dementia; C9orf72, pathogenic mutation in open reading frame 72 on chromosome 9; MAPT, pathogenic mutation in microtubule-associated protein tau; PNFA, progressive nonfluent aphasia; SD, semantic dementia.
